# Moxibustion for diarrhea in COVID-19: A protocol for systematic review and meta-analysis

**DOI:** 10.1097/MD.0000000000028777

**Published:** 2022-02-11

**Authors:** Ningning Liu, Yingxue Xu, Dongbin Zhang, Lianzhu Wang, Yi Hou, Jiafu Ji

**Affiliations:** a Affiliated Hospital of Shandong University of Traditional Chinese Medicine, Jinan, Shandong, China; b Shandong University of Traditional Chinese Medicine, Jinan, Shandong, China.

**Keywords:** COVID-19, diarrhea, meta-analysis, moxibustion, protocol

## Abstract

**Background:**

Coronavirus disease 2019 (COVID-19) is an acute respiratory infectious disease that is often accompanied by diarrhea, patients with symptoms such as diarrhea are more likely to develop severe pneumonia, while diarrhea is the most prominent among atypical symptoms. The incidence of diarrhea in COVID-19 patients is *2.0% to 49.5%.* Moxibustion has been proven to have a therapeutic effect on diarrhea; however, there is no high-quality evidence on moxibustion for diarrhea in COVID-19 patients. This study was designed to evaluate the effectiveness and safety of moxibustion for the treatment of diarrhea in patients with COVID-19.

**Methods:**

Randomized controlled trials from December 2019 to December 2021 will be included without restrictions on language or publication date. PubMed, EMBASE, Cochrane Library, Web of Science, Chinese Biomedical Databases, China National Knowledge Infrastructure, Wanfang database, and VIP database will be searched. Two researchers will independently select studies, extract data and evaluate study quality. Cochrane risk of bias tool for randomized trials will be used to assess the risk of bias of included studies. Statistical analyses will be performed using the Review Manager V.5.3 and stata 14.0.

**Results:**

The results of this meta-analysis will be submitted to a peer-reviewed journal for publication.

**Conclusion:**

This study will provide evidence for whether moxibustion therapy is beneficial to the treatment of diarrhea in COVID-19.

**Ethics and dissemination:**

Ethical approval is not required for this study. The systematic review will be published in a peer-reviewed journal, presented at conferences, and shared on social media platforms. This review would be disseminated in a peer-reviewed journal or conference presentations.

**Prospero registration number:**

CRD42022302933.

## 1 Introduction

Coronavirus disease 2019 (COVID-19) is caused by severe acute respiratory syndrome coronavirus 2 (SARS-CoV-2) infection with high infectivity and high mortality, and it has rapidly developed into a public health emergency worldwide. Apart from respiratory symptoms, gastrointestinal manifestations are common in patients with SARS, MERS, and COVID-19. We previously reported a high prevalence of enteric symptoms in patients with SARS and demonstrated acute viral replication in the small intestinal mucosa of patients with SARS. Clinical studies have shown that the incidence of diarrhea ranges from 2% to 50%. This can precede or cause respiratory symptoms. The pooled analysis revealed an overall percentage of diarrhea onset of 10.4%. SARS-CoV uses angiotensin-converting enzyme 2 (ACE2) and serine protease TMPRSS2 for S protein priming. ACE2 and TMPRSS2 are not only expressed in the lungs but also in the small intestinal epithelia. ACE2 is expressed in the upper esophagus, liver, and colon. SARS-CoV-2 binding affinity to ACE2 is significantly higher (10-20 times) than that of SARSCoV. Several reports indicate that viral RNA shedding in stool is detectable for a longer period than in nasopharyngeal swabs.^[1]^ Fecal shedding of SARS-CoV RNA was found in 86% to 100% of patients during days 6 to 14 of illness and could persist for >30 days of illness. Enteric manifestations of SAR-CoV2 not only pose important diagnostic challenges to clinicians when facing patients with mild COVID-19 symptoms at initial presentation, but also signify potential fecal transmission of this virus. With the increasing number of reported cases of COVID-19, there is a pressing need to systemically summarize the enteric 7 manifestations of COVID-19 and the temporal pattern of fecal shedding of the SARS-CoV-2 virus, particularly to gastroenterologists and endoscopists who may not be familiar with this disease.^[2]^ The disease is often treated with oral drugs, but the symptoms easily or intermittently after drug withdrawal are difficult to cure, which affects the quality of life of patients.^[3]^ Many patients are looking for complementary therapies that may be effective and are less likely to have side effects.^[4]^ Moxibustion-related interventions are among the most frequently sought complementary and alternative medicine modalities and have been widely used in various conditions, including functional gastrointestinal disorders, with 12 million treatments per year in the United States.^[5–8]^

As one of the external treatment methods of traditional Chinese medicine, moxibustion has the functions of warming meridians, dispelling cold, warming yang, stopping diarrhea, activating blood circulation, and dredging collaterals. Moxibustion improves general health and treats chronic diseases such as arthritis and digestive system disorders by using the thermal stimulation produced by burning herbal preparations containing dried *Artemisia argyi* leaves or *A argyi* leaves on acupoints. Some studies have shown that moxibustion has a unique effect on diarrhea, which can significantly improve the symptoms and signs of patients. Animal experiments have shown that moxibustion can reduce serum inflammatory factors and improve intestinal dysfunction in a rat model of diarrhea.^[9]^ At the present stage, moxibustion has been proven to participate in the treatment of COVID-19 as an auxiliary means and has achieved good results.^[10,11]^ But, there is no high-quality evidence on moxibustion for diarrhea in COVID-19. Therefore, we designed this study to better understand the effectiveness and safety of moxibustion therapy for diarrhea in patients with COVID-19.

## 2 Methods

### 2.1 Study registration

This systematic review protocol was registered with PROSPERO (No. CRD42022302933). We will follow the recommendations outlined in the Cochrane Handbook of Systematic Review of Interventions and the preferred reporting items for systematic reviews and meta-analysis protocols (PRISMA-P) statement guidelines. If amendments are required, we will update our protocol to include any changes in the entire research process.

### 2.2 Types of studies

Randomized controlled trails will be included, without restrictions on language or publication date.

### 2.3 Types of participants

Subjects with documented COVID-19 with diarrhea lasting 4 weeks or longer. There were no restrictions on sex, race, or disease stage. Patients with a history of diarrhea prior to the COVID-19 infection were excluded.

### 2.4 Types of interventions and comparisons

In addition to the treatment of COVID-19, treatment group interventions comprised moxibustion, herb partitioned moxibustion, moxibustion with amugwort stick, ormoxa cone moxibustion, and comparator groups intervention: comfort therapy (placebo, pseudo-acupunc-ture or blank control), other therapies (Western medicine, usual care or nondrug therapy, etc).

### 2.5 Types of outcomes

Outcomes include effectiveness indicators and safety indicators.

#### 2.5.1 Effectiveness indicators

Clinical variables, such as the Bristol score, number of bowel movements, quality of life, and associated symptoms of diarrhea.

#### 2.5.2 Safety indicator

The incidence of adverse events.

### 2.6 Search methods for identification of studies

Randomized controlled trials will be extracted from PubMed, EMBASE, Cochrane Library, Web of Science, Chinese Biomedical Databases, China National Knowledge Infrastructure, Wanfang Database, and VIP Database. The complete PubMed search strategy is summarized in Table 1.

**Table 1:** PubMed search strategy.

Number	Search items
#1	“covid 19”[Title/Abstract] OR “2019-nCoV”[Title/Abstract] OR “coronavirus disease 19”[Title/Abstract] OR “2019 novel coronavirus”[Title/Abstract] OR “coronavirus disease 2019”[Title/Abstract] OR “disease 2019 coronavirus”[Title/Abstract] OR “sars coronavirus 2 infection”[Title/Abstract] OR “SARS-CoV2”[Title/Abstract]
#2	“Moxibustion”[title/abstract] or “Moxibustion therapy”[title/abstract] or “herb partitioned moxibustion”[title/abstract] or “moxibustion with amugwort stick”[title/abstract] or “moxa cone moxibustion” [title/abstract]or “direct moxibustion”[title/abstract]or “indirect moxibustion”[title/abstract].
#3	“Diarrhea”[Title/Abstract] OR “functional diarrhea”[Title/Abstract] OR “chronic diarrhea”[Title/Abstract] OR “Wugeng diarrhea”[Title/Abstract]
#4	#1 and #2 and #3

### 2.7 Data collection

#### 2.7.1 Selection of studies

**Figure 1:**
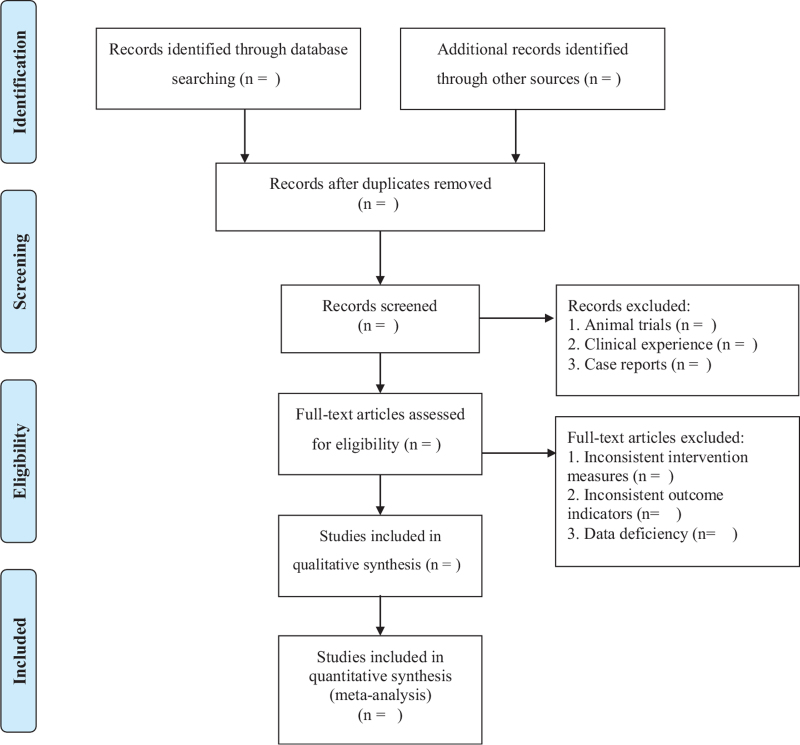
PRISMA flow chart. PRISMA = preferred reporting items for systematic reviews and meta-analysis.

Two reviewers (NNL and JFJ) will search the study independently, and then they will screen the studies by reviewing titles and abstracts or the full text if necessary. Further, unresolved discrepancies were managed by a third reviewer (LZW). The selection process was summarized using the PRISMA flow diagram. Details of the selection procedure for the studies are shown in the PRISMA flow chart (Fig. 1).

#### 2.7.2 Data extraction and management

Data were extracted from the selected studies by 2 reviewers (YXX and DBZ). The detailed extraction information was as follows: basic information of the included studies, baseline characteristics of the subjects, intervention measures and control measures, key elements of bias risk assessment, and outcome indicators. Any disagreements were resolved through discussion or consultation with a third author (YH).

EndNote X9.3 (Toronto, ON, Canada) will be used to manage the search results and perform screening, and duplicate publications will be excluded.

#### 2.7.3 Quality and bias assessment

The methodological quality and risk of bias of each trial will be independently assessed by 2 review authors (NNL and DBZ) according to the Cochrane Handbook. The following characteristics will be assessed: random sequence generation, allocation concealment, blinding of participants and personnel, blinding of outcome assessment, incomplete outcome data, selective reporting, and other bias. Based on the assessments of the studies against these 7 domains, they will be classified as being of “low risk”, “high risk”, or “unclear risk” of bias. Any disagreements were resolved through discussion or consultation with another reviewer (LZW).

#### 2.7.4 Dealing with missing data

If complete literature or relevant data are not available, the corresponding author will be contacted. However, if missing data could not be obtained, the study was excluded from the analysis.

### 2.8 Statistical analysis

#### 2.8.1 Measures of treatment effect

Review Manager (RevMan 5.3; Cochrane Collaboration, Nordic Cochrane Center, Copenha-gen, Denmark) software will be used to conduct this meta-analysis.

Dichotomous outcomes are presented as risk ratios with 95% confidence intervals. When continuous outcomes existed, the mean differences or standardized mean differences were calculated.

#### 2.8.2 Assessment of heterogeneity

The choice of whether to conduct a meta-analysis and which model to use (fixed or random effects) will depend on the level of statistical heterogeneity assessed by the *P* value and *I*^*2*^ index. *P* < .05 considered to represent significant statistical heterogeneity, and *I*^*2*^ >50% considered to be indicative of substantial heterogeneity.

#### 2.8.3 Data synthesis

The fixed effects model was used if no significant heterogeneity was observed; otherwise, the random effects model was applied for statistical analysis.^[12]^

#### 2.8.4 Subgroup analysis

According to the results of the data synthesis, we will perform subgroup analyses or a meta-regression to analyze the source of any heterogeneity.

#### 2.8.5 Assessment of reporting bias

When outcomes include more than 10 studies, we will use Stata 14.0 (Stata Corp., CollegeStation, Texas, USA) to assess the reporting bias by funnel plot and Egger test.^[13]^

#### 2.8.6 Sensitivity analysis

Sensitivity analyses will be performed to determine whether the results are affected by leave1out with Stata14.0.

#### 2.8.7 Quality of evidence evaluation

The evidence quality will be evaluated by 2 viewers (NNL and YH) independently with the grading of recommendation assessment, development, and evaluation. According to 5 parameters (publication bias, indirectness, inconsistency, imprecision, and study limitations), evidence quality will be rated “high”, “moderate”, “low” according to the rating standards.

#### 2.8.8 Ethics and dissemination

Since this study did not involve patient privacy, ethical approval was not required. Our research results will be shared and presented through conference reports and peer-reviewed journals.

## 3 Discussion

COVID-19 caused by SARS-CoV-2, which has become a serious public health threat worldwide, with millions of people at risk in increasing countries.^[14]^ Dyspnea, fatigue, diarrhea, and abdominal pain are highly prevalent in COVID-19; however, the COVID-19 pandemic has resulted in numerous patients complaining of digestive system symptoms, such as diarrhea.^[15]^ Diarrhea can lead to the worsening of symptoms and intensification of negative emotions.

Moxibustion, a very ancient modality of treating diseases, has been used throughout the history of human civilization and plays an important role in disease resistance. Moxibustion has been widely used for various conditions, including cancer, ulcerative colitis, stroke rehabilitation, constipation, hypertension, pain conditions, and breech presentation. It is benefit for COVID-19 patients with diarrhea to accept acupuncture treatment. Thus, this study aimed to provide evidence of acupuncture therapy for diarrhea in COVID-19 and aid treatment decisions.

## Author contributions

**Data collection:** Yingxue Xu and Yi Hou.

**Funding support:** Jiafu Ji.

**Resources:** Jiafu Ji.

**Software operating:** Dongbin Zhang and Lianzhu Wang.

**Supervision:** Jiafu Ji.

**Writing – original draft:** Ningning Liu and Jiafu Ji.

**Writing – review & editing:** Ningning Liu and Jiafu Ji.
